# Annatto Tocotrienol Improves Indices of Bone Static Histomorphometry in Osteoporosis Due to Testosterone Deficiency in Rats

**DOI:** 10.3390/nu6114974

**Published:** 2014-11-10

**Authors:** Kok-Yong Chin, Saif Abdul-Majeed, Nur Farhana Mohd. Fozi, Soelaiman Ima-Nirwana

**Affiliations:** Department of Pharmacology, Faculty of Medicine, Universiti Kebangsaan Malaysia, Jalan Yaacob Latif, Bandar Tun Razak, Cheras, 56000 Kuala Lumpur, Malaysia; E-Mails: gabrielyong@live.com.my (K.-Y.C.); SaifSaad@imu.edu.my (S.A.-M.); daisy_farhana@yahoo.com (N.F.M.F.)

**Keywords:** osteoblast, osteoclast, osteoporosis, testosterone, tocotrienol, vitamin E

## Abstract

This study aimed to evaluate the effects of annatto tocotrienol on indices of bone static histomorphometry in orchidectomized rats. Forty male rats were randomized into baseline (BL), sham (SH), orchidectomized (ORX), annatto tocotrienol-treated (AnTT) and testosterone enanthate-treated (TE) groups. The BL group was sacrificed upon receipt. All rats except the SH group underwent bilateral orchidectomy. Annatto tocotrienol at 60 mg/kg body weight was administered orally daily to the AnTT group for eight weeks. Testosterone enanthate at 7 mg/kg body weight was administered intramuscularly once weekly for eight weeks to the TE group. The rat femurs were collected for static histomorphometric analysis upon necropsy. The results indicated that the ORX group had significantly higher osteoclast surface and eroded surface, and significantly lower osteoblast surface, osteoid surface and osteoid volume compared to the SH group (*p* < 0.05). Annatto tocotrienol and testosterone enanthate intervention prevented all these changes (*p* < 0.05). The efficacy of annatto tocotrienol was on par with testosterone enanthate. In conclusion, annatto tocotrienol at 60 mg/kg can prevent the imbalance in bone remodeling caused by increased osteoclast and bone resorption, and decreased osteoblast and bone formation. This serves as a basis for the application of annatto tocotrienol in hypogonadal men as an antiosteoporotic agent.

## 1. Introduction

Osteoporosis is a metabolic bone disease characterized by decreased bone density and degeneration of bone microarchitecture. Male osteoporosis is not as prevalent as female postmenopausal osteoporosis [1]. However, male osteoporotic patients suffer from greater mortality and morbidity compared to their female counterpart [2,3]. Testosterone deficiency is the most common cause of male osteoporosis [4]. Testosterone deficiency elicits an imbalance in bone remodeling, whereby bone formation is decreased and bone resorption is increased, resulting in a net bone loss [5]. The standard antiosteoporotic therapies for male hypogonadal osteoporosis such as testosterone therapy, bisphosphonates and teriparatide are effective in increasing bone mineral density and preventing fractures, but they are not free from side effects [6,7,8].

Tocotrienol and tocopherol constitute the vitamin E family. Both groups contain a chromanol ring and a long carbon tail [9,10]. The presence of three double bonds on the carbon tail of tocotrienol separates it from tocopherol [9,10]. Both groups can be further divided into α-, β-, γ- and δ- isomers based on the position of the side chain on the chromanol ring [9,10]. These vitamin E isomers usually exist as a mixture in natural sources [9,10]. Gamma-tocotrienol is the predominant vitamin E isomer in palm oil [11] while delta-tocotrienol is the most abundant isomer in annatto bean [12].

Previous studies have revealed that tocotrienol demonstrates potential antiosteoporotic effects in various animal models [13,14,15], but the skeletal effects of tocotrienol on osteoporosis induced by testosterone deficiency have not been fully explored. A study by Ima-Nirwana *et al.* (2000) [16] has shown that palm tocotrienol can prevent the decrease in bone mineral density due to testosterone deficiency in rats. Recently, annatto tocotrienol has been shown to improve bone structural and dynamic indices at the femur of orchidectomized rats [17]. The effect of tocotrienol on indices of bone static histomorphometry, which quantify the number of osteoblasts and osteoclasts which govern bone remodeling, bone erosion and osteoid deposition [18], in osteoporosis due to testosterone deficiency is not well established.

Tocotrienol mixture derived from palm oil has been the focus of previous studies on the antiosteoporotic effects of tocotrienol [14,15]. Palm tocotrienol contains a substantial amount of alpha-tocopherol [11]. Alpha-tocopherol has been reported to attenuate the antiosteoporotic effects of tocotrienol by decreasing the bioavailability of tocotrienol [19]. Alpha-tocopherol in large doses may also promote osteoclast fusion and differentiation, and subsequently causing adverse effects on bone health [20]. This property is not shared by tocotrienol isomers [20]. Annatto tocotrienol mixture, which does not contain alpha-tocotrienol, renders an opportunity to study the bone protective effects of tocotrienol without the interference of alpha-tocopherol.

The objective of the current study was to evaluate the effects of annatto tocotrienol at 60 mg/kg body weight on static histomorphometric indices in testosterone deficiency model in rats. Abdul-Majeed *et al.* (2012) [21] have shown that annatto tocotrienol at 60 mg/kg body weight for eight weeks can improve the levels of bone remodelling markers and static histomorphometric indices in ovariectomized rats. Hence, we hypothesized that annatto tocotrienol at 60 mg/kg for eight weeks could prevent degenerative changes in bone static indices in osteoporosis due to testosterone deficiency. We hope the evidence provided by this study can serve as a basis for the application of annatto tocotrienol in preventing male osteoporosis.

## 2. Materials and Methods

### 2.1. Preparation of Treatment 

Annatto tocotrienol, which contained 90% delta-tocotrienol and 10% gamma-tocotrienol, was a gift from American River Nutrition Inc. (Hadley, MA, USA). It was diluted in olive oil (Bartolini Emilio, Arrone Terni, Italy) before given to the rats so that the delivery volume did not exceed 1 mL/100 g body weight. Olive oil was used as the vehicle because its vitamin E content was low (51 ppm) [16,22]. Testosterone enanthate (Jesalis Pharma, Jena, Germany) was diluted in peanut oil (Sime Darby, Subang Jaya, Malaysia). Peanut oil was used as the vehicle because the original formulation was suspended in peanut oil.

### 2.2. Animals and Treatment

Forty male Sprague-Dawley rats aged three months old weighing 250–300 g were acquired from the Laboratory Animal Research Unit of Universiti Kebangsaan Malaysia. The rats were housed one per plastic cage in the vivarium at the Department of Pharmacology, Universiti Kebangsaan Malaysia under standard condition (natural light cycle and ambient temperature 25–27 °C). The rats were given standard diet (Gold Coin, Klang, Malaysia) and tap water *ad libitum*. One week of acclimatization was allocated before the rats received surgical treatment. The rats were randomized into five groups, which were the baseline (BL), sham (SH), orchidectomized (OVX), annatto tocotrienol-treated (AnTT) and testosterone enanthate-treated (TE) groups. The BL group was sacrificed at the beginning of the study. The OVX, AnTT and TE groups underwent bilateral orchidectomy. The SH group underwent similar surgical stress but their testes were not removed. The rats received their respective treatment after one week of recuperation. The AnTT group received 60 mg/kg body weight annatto tocotrienol orally daily for eight weeks while the other groups received equivolume of olive oil. The TE group received 7 mg/kg body weight testosterone enanthate intramuscular injection once weekly while the other groups received equivolume of peanut oil. Body weight of the rats was measured using a weighing scale (Tanita, Arlington Heights, IL, USA) and was recorded weekly. Blood was collected at the start and the end of the treatment period using plain tubes. Serum was extracted immediately and stored in −70 °C until analyzed. All rats were sacrificed at the end of the treatment period and their femurs were harvested and kept in 10% formalin for bone static histomorphometric analysis.

### 2.3. Ethical Consideration

The protocol of this study was reviewed and approved by Universiti Kebangsaan Malaysia Animal Ethical Committee (Approval Code: FP/FAR/2012/IMA/18-JULY/445-JULY-2012-APRIL-2014).

### 2.4. Bone Remodeling Markers

Enzyme-linked immunosorbent assay (IDS, Tyne & Wear, UK) was used to determine the *N*-terminal propeptide of type I procollagen (PINP) (bone formation marker) and osteoclast-derived tartrate-resistant acid phosphatase form 5b (TRACP 5b) levels in rat serum. The assays were performed as per procedures described by the manufacturer.

### 2.5. Bone Cellular Histomorphometric Analysis

The distal left femur was sawn in half and was decalcified using ethylenediaminetetraacetic acid for three months. After the bone was decalcified, it was embedded in paraffin wax. The bone samples were sectioned with a microtome (Leica, Wetzlar, Germany) at a thickness of 5 μm. The sections were stained with haematoxylin and eosin.

Micrographs of the bone sections were taken using a microscope (Nikon Eclipse 80i, Chiyoda, Japan) and an image analyzer (MediaCybernetics Image Pro-Plus, Rockville, MD, USA) at a magnification of 300×. Assessment of bone static histomorphometric parameters were performed by a blinded examiner using Weibel Grid technique. The static parameters measured included osteoblast surface (ObS/BS), osteoclast surface (OcS/BS), eroded surface (ES/BS), osteoid surface (OS/BS) and osteoid volume (OV/BV). The region of interest was the secondary spongiosa of the metaphysis located 1 mm from the lateral cortex and 3–7 mm below the epiphyseal plate. The procedure of bone histomorphometry had been described elsewhere [21].

### 2.6. Statistical Analysis

Normality of the data was determined using Kolmogorov-Smirnov test. All data were normally distributed. Comparisons of the percentage difference in bone remodeling marker levels (between pre- and post-treatment) and the bone static parameters among the groups were performed using one-way analysis of variance (ANOVA) with post-hoc pairwise comparison. The data were expressed in mean (standard error of mean). Significant value was set at *p* < 0.05. Statistical analysis was performed using Statistical Package for Social Sciences version 21 (IBM, Armonk, NY, USA).

## 3. Results

The levels of PINP and TRACP 5b decreased after eight weeks regardless of treatment. The rates of decrease for both bone remodeling markers were the smallest in the ORX group compared to the other group but the difference was not significant (*p* > 0.05). Neither annatto tocotrienol nor testosterone enanthate treatment caused significant changes on the rate of decrease in both markers (*p* > 0.05) (Table 1).

Orchidectomy caused significant increases in osteoclast surface and eroded surface, and significant decreases in osteoblast surface, osteoid surface and osteoid volume in the ORX group compared to the SH group (*p* < 0.05). Both annatto tocotrienol and testosterone enanthate treatments prevented these changes completely (*p* < 0.05 compared to ORX group). There were no significant differences in indices of bone static histomorphometry between the AnTT and TE groups (*p* > 0.05) (Table 2).

**Table 1:** Percentage difference in the level of bone remodeling markers before and after treatment.

Groups	% difference in PINP *		% difference in TRACP 5b *	
	mean	SEM	mean	SEM
SH	−58.92	7.95	−53.36	2.20
ORX	−66.05	5.30	−44.45	5.64
AnTT	−71.20	5.31	−49.08	2.46
TE	−74.60	4.34	−56.59	3.15

* percentage difference = (post-treatment level – pre-treatment level)/pre-treatment level × 100%; *Abbreviation*: SH = sham group; ORX = orchidectomized group; AnTT = annatto tocotrienol-treated group; TE = testosterone enanthate-treated; PINP= *N*-terminal propeptide of type I procollagen; TRACP 5b = osteoclast-derived tartrate-resistant acid phosphatase form 5b, SEM = standard error of mean.

**Table 2:** Indices of bone static histomorphometry among the study groups.

Groups	Osteoblast Surface		Osteoclast Surface		Eroded Surface		Osteoid Surface		Osteoid Volume	
	mean	SEM	mean	SEM	mean	SEM	mean	SEM	mean	SEM
BL	28.46	1.41	35.21	3.15	48.56	2.66	35.16	2.40	19.04	1.08
SH	36.47	4.36	7.04 ^a^	0.78	17.49 ^a^	4.30	42.51	3.12	17.83	0.53
ORX	12.80 ^ab^	1.00	40.97 ^b^	4.01	63.97 ^ab^	1.87	12.62 ^ab^	1.84	6.20 ^ab^	0.86
AnTT	50.88 ^ac^	4.03	9.57 ^ac^	0.50	5.22 ^ac^	0.88	50.89 ^ac^	3.89	23.40 ^c^	2.36
TE	46.44 ^ac^	3.20	11.93 ^abc^	0.99	8.88 ^ac^	0.43	46.63 ^ac^	2.12	25.77 ^bc^	1.71

Letters indicate significant differences between the marker groups and (a) baseline; (b) sham; (c) ovariectomized groups. *Abbreviation*: BL = baseline group; SH = sham group; ORX = orchidectomized group; AnTT = annatto tocotrienol-treated group; TE = testosterone enanthate-treated; SEM = standard error of mean.

## 4. Discussion

The current study found that testosterone deficiency caused an increase in osteoclast surface and a reduction in osteoblast surface at the trabecular bone. This caused an imbalance in bone remodeling, which resulted in increased erosion, indicated by elevated eroded surface, and a decrease in matrix synthesis, indicated by reduced osteoid surface and volume. This would weaken the bone and subsequently causing osteoporosis. However, the imbalance in bone remodeling was not reflected in the percentage difference in bone remodeling marker levels. These changes might be transient or cycling, thus not detected in this study. Another possibility might be that the changes in bone remodeling marker levels had subsided over two months and it could be have been detected at earlier time point. The osteoclast number and eroded surface were significantly higher in the baseline group compared to the sham group. The observation might reflect a higher bone remodeling rate in the baseline group (three months old) as compared to the sham group (five months old). This was supported by higher pre-treatment (three months old) levels of serum bone remodeling markers compared to post-treatment (five months old) levels across all groups. 

Annatto tocotrienol supplementation was as effective as testosterone enanthate intervention in suppressing the adverse effects of testosterone deficiency on bone. This was evidenced by significant improvements in bone static parameters in the AnTT and TE groups as compared to the ORX group. A previous study showed that supplementation with annatto tocotrienol (10% gamma-tocotrienol and 90% delta-tocotrienol) at 60 mg/kg body weight in orchidectomized rats for eight weeks significantly improved structural histomorphometric indices at the femur [17]. Another study supplementing 30 mg/kg body weight palm vitamin E (24.4% alpha-tocopherol, 21.6% alpha-tocotrienol, 27.7% gamma-tocotrienol, 11.0% delta-tocotrienol, 15.3% palm olein) in orchidectomized rats for eight months showed improvement in bone mineral density at various skeletal sites [16]. The current findings suggested that the ability of tocotrienol to halt the decrease in osteoblast number and the increase in osteoclast number due to testosterone deficiency accounted for the improvements in bone health shown in previous studies.

Previous studies supplementing individual vitamin E isomers on normal male rats for two months demonstrated that tocotrienol, especially gamma-tocotrienol improved structural, dynamic and static histomorphometry indices in the treated group as compared to the sham group [23,24]. This indicated that the bone enhancing effects of tocotrienol was manifested with and without the onset of hypogonadal osteoporosis. The annatto tocotrienol used in this study consisted of 90% delta-tocotrienol and 10% gamma-tocotrienol. These two individual isomers were shown by Mehat *et al.* (2010) [23] to possess prominent bone anabolic effects. In an *in vitro* study by Brooks *et al.* (2011) [25], gamma- and delta-tocotrienol were shown to suppress osteoclast formation from human peripheral blood mononuclear cells. In both studies, the biological activity of gamma-tocotrienol was higher than delta-tocotrienol [23,25].

Tocotrienol also inhibited adverse changes in bone static parameters in other animal models of osteoporosis. Hermizi *et al.* (2009) [26] showed that supplementation of either palm tocotrienol rich fraction (43% alpha-tocotrienol, 31% gamma-tocotrienol, 14% delta-tocotrienol, and 12% other components) or gamma tocotrienol isomer at 60 mg/kg body weight for two months prevented the increase in osteoclast surface and eroded surface in a nicotine induced osteoporosis model. Ahmad *et al.* (2005) [27] indicated that palm tocotrienol (30.7% alpha-tocotrienol, 55.2% gamma-tocotrienol, 14.2% delta-tocotrienol) supplemented rats showed increased osteoblast number and decreased eroded surface, osteoid surface and volume compared to unsupplemented rats in a free radical-induced osteoporosis model after eight weeks. Muhammad *et al.* (2012) [28] demonstrated that pure palm tocotrienol (37.2% alpha-tocotrienol, 39.1% gamma-tocotrienol, 22.6% delta-tocotrienol, <0.5% alpha-tocopherol) at 60 mg/kg for four weeks decreased osteoclast surface but not osteoblast surface in rats with osteoporosis induced by estrogen deficiency. Abdul-Majeed *et al.* [21] showed that annatto tocotrienol (10% gamma-tocotrienol and 90% delta-tocotrienol) at 60 mg/kg for eight weeks prevented adverse changes in all static parameters due to ovariectomy in rats.

There are several possible mechanisms by which tocotrienol exerts its antiosteoporotic effects. Tocotrienol is a strong antioxidant [29]. It can protect osteoblast from oxidative damage and suppress free-radical induced osteoclast formation [15,30]. It is also a down-regulator of the mevalonate pathway, inhibition of which leads to increased osteoblast proliferation and bone formation and decreased osteoclast proliferation and survival [13,31]. It also suppresses the expression of proinflammatory cytokines, such as interleukin-1 and interleukin-6, which are known to promote osteoclastogenesis [32,33]. Previous studies also showed that tocotrienol could up-regulate gene expression related to bone formation, such as those coding for alkaline phosphatase, beta-catenin, collagen 1 alpha 1, runt related factor-2 and osterix [17,34]. These possible mechanisms should be examined in detailed in future studies.

The static indices of the AnTT group were on par with the TE group, indicating that tocotrienol was as effective as testosterone enanthate in preventing the imbalance in bone remodelling due to testosterone deficiency. We prudently suggest that annatto tocotrienol can be used together with or replace standard antiosteoporotic agents. Prolonged testosterone administration could lead to prostate cancer and cardiovascular diseases [7]. Previous toxicological evaluation found that tocotrienol demonstrated adverse effects on coagulation profile in mice at 500 and 1000 mg/kg body weight [35]. Based on the Reagan-Shaw formula [36], these translated to 250 and 500 mg/kg body weight in rats. Hence, tocotrienol at 60 mg/kg body weight as applied in this study was well below the toxic dose and was safe to be used. However, clinical trials should be performed to evaluate the effectiveness of tocotrienol in preventing osteoporosis in human. 

Several limitations need to be addressed in this study. Only a single dose of tocotrienol (60 mg/kg body weight) was tested in this study. The skeletal effects of tocotrienol at higher doses are not known. This dose was chosen because it was proven effective in many previous studies using different models of osteoporosis [14]. Group treated with tocotrienol mixtures containing alpha-tocopherol like palm vitamin E was not established in the current study. Therefore, the difference in the efficacy between tocotrienol mixtures with or without alpha-tocopherol in preventing bone loss could not be assessed. The tocotrienol level in the blood and in the bone of the rats was not measured. Hence, we do not know the bioavailability and pharmacokinetics of tocotrienol in the bone. The antiosteoporotic mechanism of tocotrienol was not determined in this study. An *in vitro* assessment of annatto tocotrienol on bone cells should be performed to illustrate the mechanisms.

This is the first time the effects of tocotrienol on static histomorphometric indices were evaluated in a testosterone deficiency model in rats. The composition of annatto tocotrienol used is unique, in which the predominant vitamin E isomer is delta-tocotrienol and it does not contain alpha-tocopherol. In comparison with most studies using palm tocotrienol, which is rich in gamma-tocotrienol, this study demonstrated that annatto tocotrienol could also achieve its antiosteoporotic potential by inhibiting the imbalance in bone cells that govern skeletal remodeling.

## 5. Conclusions

In conclusion, annatto tocotrienol at 60 mg/kg body weight can prevent osteoporosis due to testosterone deficiency as evidenced by improved static histomorphometric indices in the treated group. The efficacy of annatto tocotrienol is on par with testosterone enanthate intervention. *In vitro* studies should be conducted to illustrate the antiosteoporotic action of annatto tocotrienol. Clinical trials should be performed to validate the ability of annatto tocotrienol to prevent osteoporosis due to testosterone deficiency in humans.
